# Supplementary material for: Endometrial cancer progression driven by PTEN-deficiency requires miR-424(322)~503
Source: Cell Death Dis. 2025 Oct 6;16(1):705. doi: 10.1038/s41419-025-08022-z (PMC12501053; doi:10.1038/s41419-025-08022-z)
Supplement: Supplementary file 1 — Uncropped Western blots [file 41419_2025_8022_MOESM1_ESM.pdf]

Figure-1J

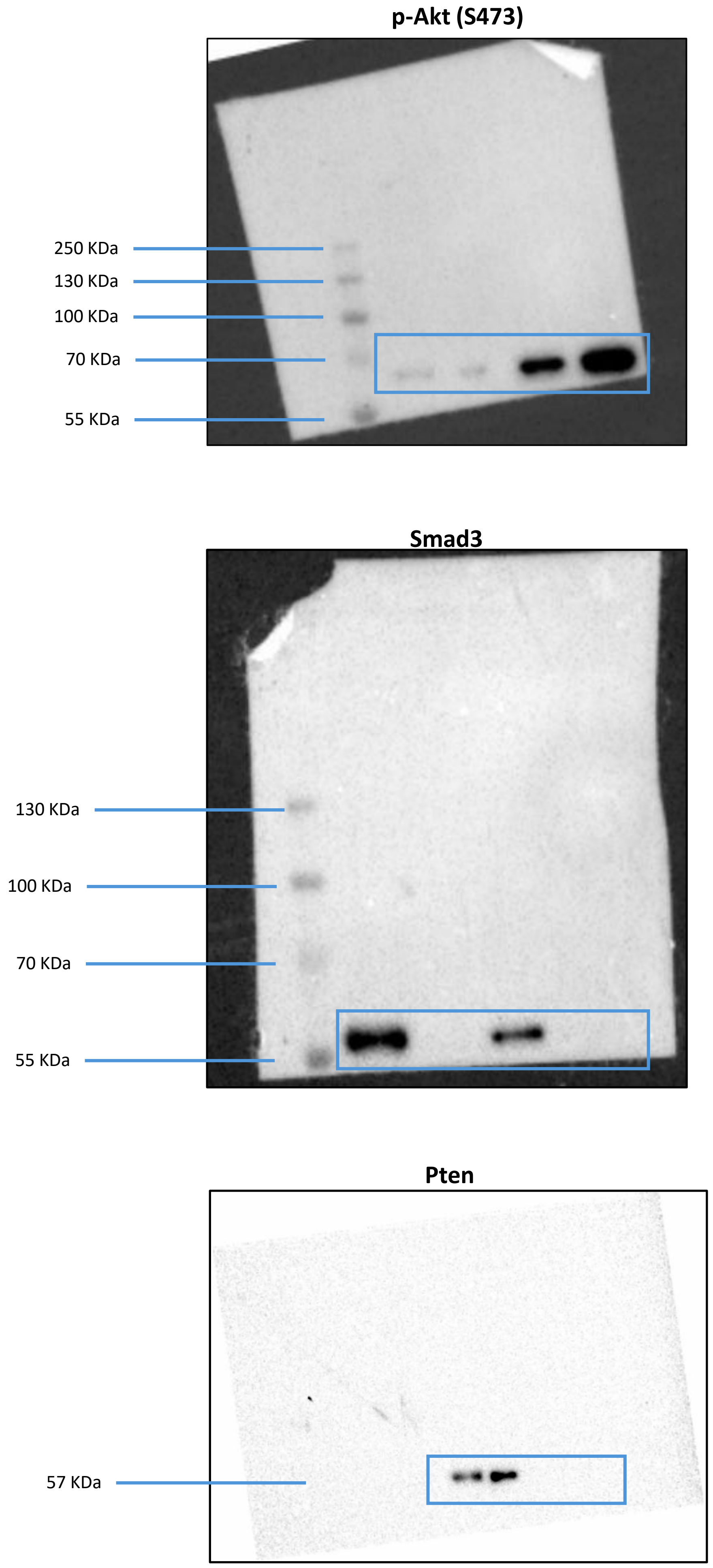
p-Akt (S473)
250 KDa
130 KDa
100 KDa
70 KDa
55 KDa
Smad3
130 KDa
100 KDa
70 KDa
55 KDa
Pten
57 KDa

Figure-1J

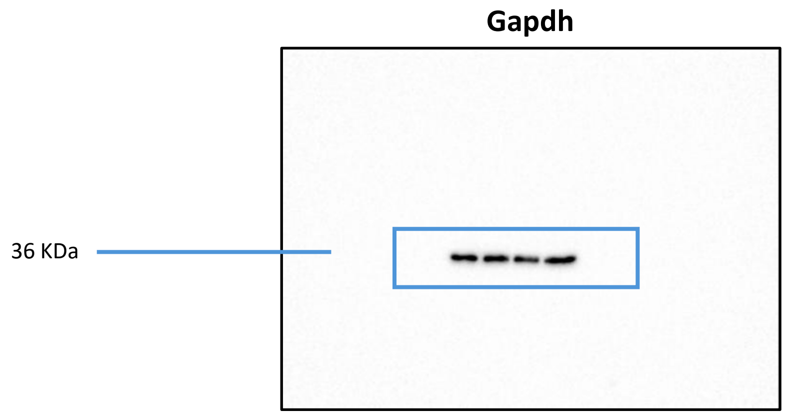

Figure-4B

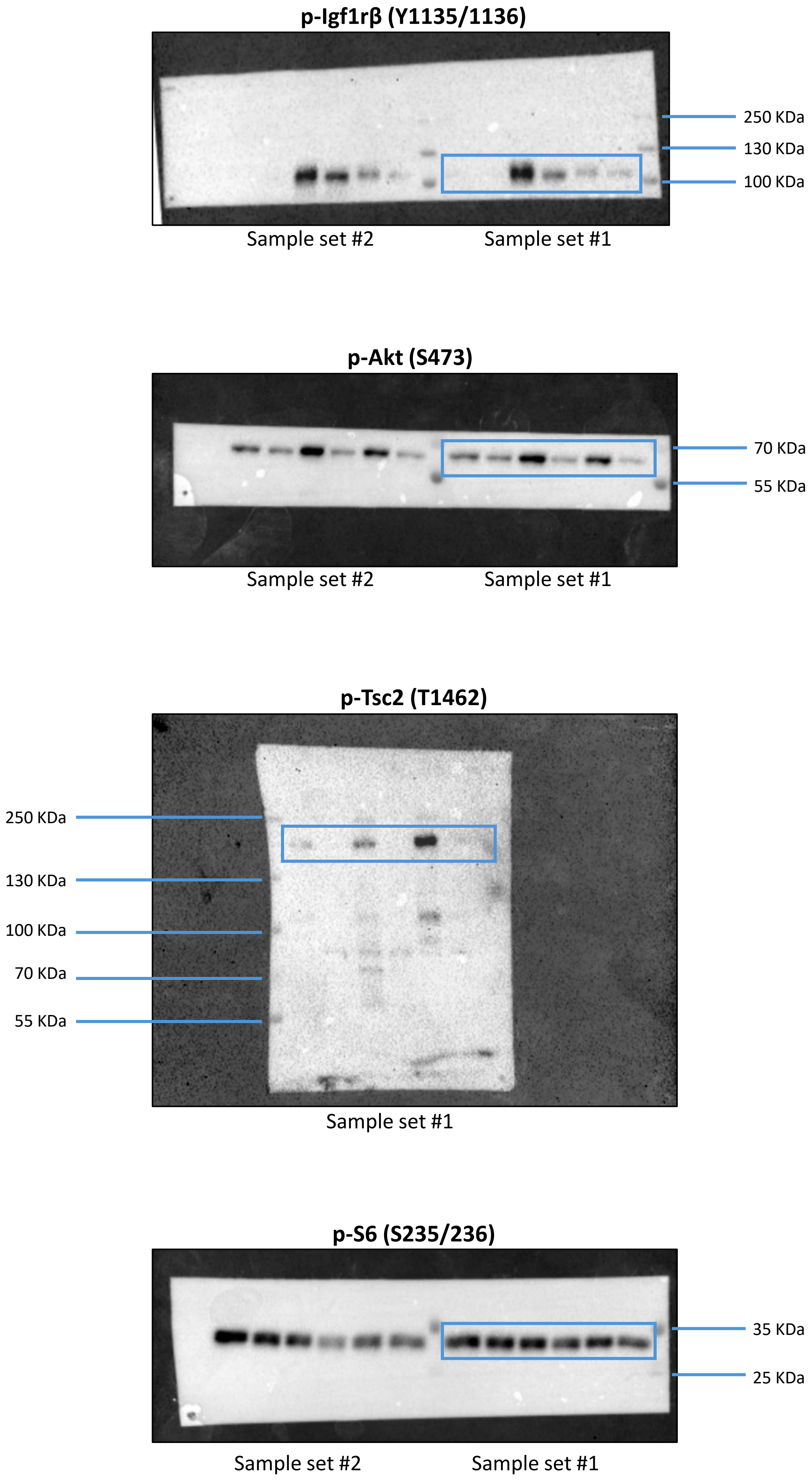
p-Igf1rβ (Y1135/1136)
250 KDa
130 KDa
100 KDa
Sample set #2
Sample set #1
p-Akt (S473)
70 KDa
55 KDa
Sample set #2
Sample set #1
p-Tsc2 (T1462)
250 KDa
130 KDa
100 KDa
70 KDa
55 KDa
Sample set #1
p-S6 (S235/236)
35 KDa
25 KDa
Sample set #2
Sample set #1

Figure-4B

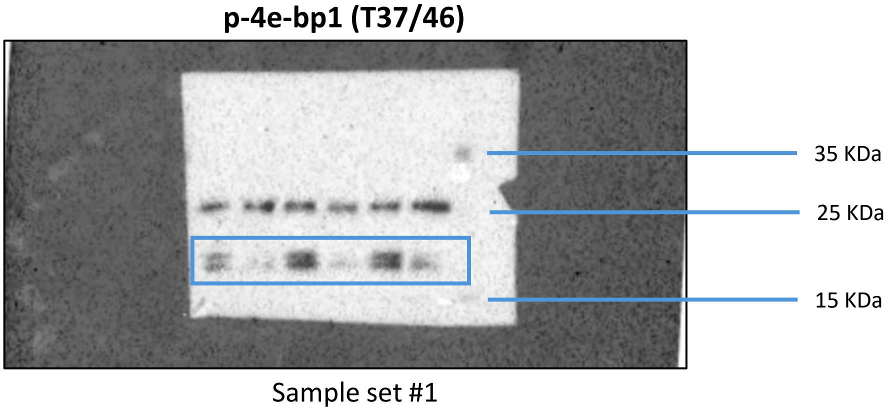
p-4e-bp1 (T37/46)
35 KDa
25 KDa
15 KDa
Sample set #1

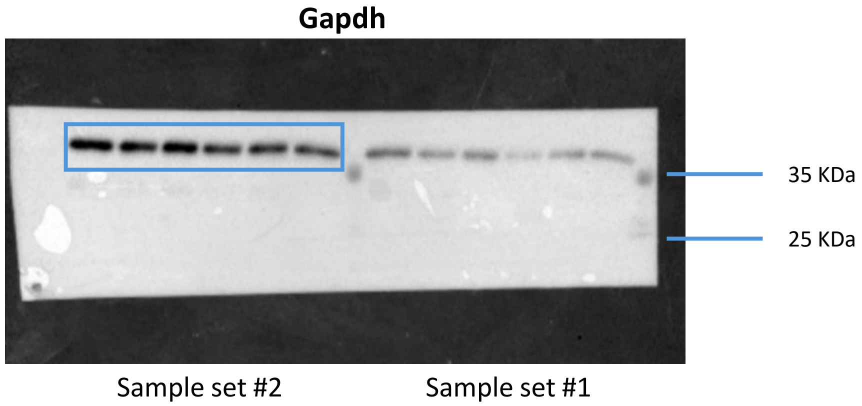
Gapdh
35 KDa
25 KDa
Sample set #2
Sample set #1
